# Assessing the Reliability, Accuracy, and Relevance of Artificial Intelligence Speech Recognition for Clinical Documentation: A Scoping Review

**DOI:** 10.1111/jep.70460

**Published:** 2026-04-29

**Authors:** Samuel Atiku, Kehinde Owolanke, Olufisayo Olakotan

**Affiliations:** ^1^ Digital Technology and Innovation University of Staffordshire England UK; ^2^ Research Services Aston University Birmingham Birmingham UK; ^3^ Department of Obstetrics and Gynaecology Mersey and West Lancashire NHS Trust Leicester UK; ^4^ Department of Neonatology, Women and Children's Directorate University Hospitals Leicester NHS Trust Leicester UK

## Abstract

**Background:**

Background Clinical documentation is a major contributor to clinician workload and burnout, with physicians spending more than half of their workday on electronic health record (EHR) tasks. Artificial intelligence (AI)–based speech recognition (ASR) tools promise to reduce this burden by generating draft notes from dictated or conversational clinical encounters. Despite rapid adoption, concerns remain about their real‐world accuracy, reliability, and ability to capture clinically relevant information.

**Aims:**

To systematically map the breadth of published evidence reporting on the accuracy, reliability, efficiency, and clinical information capture of ASR systems used in healthcare settings for clinical documentation.

**Methods:**

The scoping review employed the methodology developed by Arksey and O'Malley in 2005 and further expanded by Levac and Colquhoun 2010. Four databases (PubMed, Scopus, Web of Science, and MEDLINE) were searched for studies published between 2008 and 2025. All findings were reported according to PRISMA guidelines for scoping reviews.

**Results:**

Of 3,520 records, thirty‐two met the inclusion criteria, using benchmarking studies, controlled comparisons, qualitative methods, and retrospective reviews. Across settings, ASR showed substantial accuracy limitations, with word error rates ranging from moderate in dictated notes to very high in conversational and emergency contexts. Common errors included deletions, substitutions, and misrecognition of medication names or brief utterances. Although some studies reported reduced typing burden and improved workflow efficiency, systems frequently missed clinically relevant details. Evidence for improvements in note completeness was mixed, and little research linked system accuracy to patient safety or diagnostic outcomes.

**Conclusion:**

ASR can reduce typing and improve documentation efficiency, sometimes capturing richer narrative detail. However, frequent and clinically significant errors shaped by linguistic complexity, context, and speaker variation make unsupervised use unsafe. Human oversights remains essential, and continued refinement, rigorous evaluation, and attention to workflow, cognitive burden, and equity are required.

## 1 Introduction

Clinical documentation is an essential but time‐intensive aspect of modern health care. Many clinicians now spend more time on electronic paperwork than on face‐to‐face patient care. For example, primary care physicians devote nearly 6 h of an 11.4 h workday to electronic health record (EHR) tasks (about half their day), including roughly 1–2 h of after‐hours “pajama time” each night on documentation [1, 2]. Studies have observed that physicians in ambulatory settings spend more than half of their workday interacting with the EHR and only around 25% of their time with patients [2, 3]. This clerical burden is strongly linked to provider burnout, job dissatisfaction, and intent to leave practice, as well as to potential reductions in care quality [2, 3].

Artificial intelligence (AI) has opened new possibilities for improving clinical documentation. Artificial Inelligence (AI) based speech recognition (ASR) or Automatic speech recognition (ASR) technology enables clinicians to dictate rather than type their notes, offering a potential solution to reduce documentation burden and improve efficiency in clinical workflows [4]. ASR systems (e.g. front‐end dictation software like Dragon Medical) are already widely used; in fact, over 90% of U.S. hospitals reported plans to expand use of voice recognition for clinical documentation [4, 5]. Building on these foundations, new AI‐driven transcription tools integrate advanced speech recognition, natural language processing, and large language models to listen to clinical encounters in real time and automatically generate draft notes [6, 7]. These systems capture the patient visit, often through a microphone or smart device in the exam room, and produce an initial clinical note or summary without the clinician having to manually enter data [6, 7].

Despite the promise of AI‐driven tools such as ASR for clinical documentation, uncertainty remains about their real‐world accuracy, reliability, and how well they integrate into clinicians' workflows [8, 9]. Studies reported that while modern speech‐to‐text technology can achieve near–human‐level accuracy under optimal conditions, real‐world performance remains inconsistent, with error rates that vary widely and can be unacceptably high in clinical practice [10, 11]. A recent systematic review of AI transcription systems found reported word error rates ranging from as low as 0.1% in controlled dictation settings to over 50% in conversational, multi‐speaker scenarios [5]. One study of dictated notes from multiple hospitals found an initial 7.4% error rate (approximately seven errors per 100 words) in raw ASR generated transcripts, even when using a leading medical ASR product [4]. Another study focusing on an emergency department setting, 71% of speech‐recognized notes contained at least one error, and 15% of these notes contained a critical error that could potentially impact patient safety [11]. These findings underscore that without human oversight, AI‐generated documentation can introduce inaccuracies.

While prior studies have examined the technical accuracy of speech recognition systems in healthcare, the broader question of how these technologies perform in real‐world clinical documentation remains insufficiently synthesized [4, 11, 12, 13]. Much of the existing literature focuses on transcription performance in controlled settings or within single clinical specialties, often emphasizing technical metrics such as word error rate without examining the implications for clinical meaning, documentation completeness, workflow integration, or patient safety [4, 11, 12, 13]. Consequently, there remains limited consolidated evidence on the reliability of ASR in capturing medically relevant information during routine clinical encounters. This scoping review aims to address this gap by synthesizing evidence across diverse healthcare settings to evaluate the accuracy, reliability, clinical relevance, and workflow implications of AI‐driven ASR tools used for clinical documentation.

### 1.1 Methodology

This scoping review employs the methodology developed by Arksey and O'Malley in 2005 [14] and further expanded by Levac and Colquhoun 2010 [15]. The method involves fives key steps: identifying the research question, identifying relevant studies, selecting the studies, charting the data, collating, summarizing, and reporting the results. All findings were reported according to PRISMA guidelines for scoping reviews. A study protocol review protocol was developed a priori; however, the protocol was not formally registered.

### 1.2 Research Question

What does the published evidence report regarding the accuracy, reliability, efficiency, and clinical information capture of ASR systems used in healthcare documentation across diverse healthcare settings?

### 1.3 Identifying Relevant Studies

A comprehensive literature search was conducted to identify relevant studies published between 2008 and 2025 across four electronic databases: PubMed, Scopus, Web of science, and MEDLINE, using a search strategy that combined keywords and subject headings related to the concepts of interest (Appendix C). The search terms encompassed AI‐driven speech recognition and clinical documentation, along with terms related to accuracy, efficiency, and reliability, to ensure that studies reporting these outcomes were captured. Grey literature, conference abstracts without full‐text articles, dissertations, and preprints were excluded to ensure inclusion of peer‐reviewed empirical evidence.

### 1.4 Eligibility Criteria

We established inclusion and exclusion criteria iteratively based on our research question and evolving familiarity with the literature. We included primary studies, regardless of design (quantitative, qualitative, mixed methods), that evaluated or reported on ASR systems used in healthcare settings for clinical documentation. Studies were eligible if they reported performance‐related findings concerning accuracy, efficiency (e.g., time savings, productivity, workflow impact), reliability, or clinical information capture. Uniform outcome measures were not required due to anticipated heterogeneity in evaluation approaches across systems and settings. Studies were excluded if they did not examine an ASR tools in a clinical documentation context or if they provided no evaluation of performance‐related issues (e.g., studies focused solely on algorithm development without reporting findings from clinical application, or studies conducted outside healthcare settings).

### 1.5 Study Selection

All retrieved records were imported into reference management software and duplicates were removed. Two reviewers (OO and AS) independently screened titles and abstracts against the eligibility criteria. Articles deemed potentially relevant underwent independent full‐text review by both reviewers. Disagreements were resolved through discussion, with a third reviewer available for adjudication where consensus could not be reached. The study selection process is presented using a PRISMA‐ScR flow diagram detailing records identified, screened, excluded, and included.

### 1.6 Charting the Data

We developed a standardized data‐charting form to extract key information from each study. For each included study, we collected details on the publication, study design, setting, population, and the characteristics of the AI‐based speech recognition or scribe system. Specific outcome data such as word error rates, transcription accuracy, documentation time savings, user satisfaction, or clinically relevant error rates were recorded to address the review question. Data extraction was conducted by one reviewer and independently verified by a second reviewer to ensure accuracy and consistency, with disagreements resolved through consensus. We used an iterative approach to data charting, refining the extraction form as needed after piloting it on an initial subset of studies. This iterative refinement ensured that all relevant variables required to answer the research question were fully captured.

### 1.7 Collating, Summarizing, and Reporting the Results

We collated the extracted data and synthesised the findings using thematic analysis. Given the substantial variation in study designs, outcome measures, and reported ASR performance metrics across diverse clinical contexts, findings were synthesised narratively rather than quantitatively compared across systems. This approach enabled structured integration of heterogeneous evidence. Through this process, we identified recurring patterns and issues that were grouped thematically and presented through narrative synthesis to map the range and characteristics of reported outcomes across diverse clinical settings.

A methodological appraisal using the Mixed Methods Appraisal Tool (MMAT, 2018 version) [16]. The MMAT was selected because the included studies were methodologically heterogeneous, encompassing qualitative, quantitative (randomized, non‐randomized, and descriptive), and mixed‐methods designs. Each study was appraised using the MMAT criteria appropriate to its specific design category. The quality of each study was assessed using the MMAT checklist, and the appraisal results are presented in Appendix A. Overall, eighteen studies demonstrated high methodological quality, while the remaining fourteen were considered to have acceptable methodological quality. All studies were retained in the review.

## 2 Results

**Figure 1:**
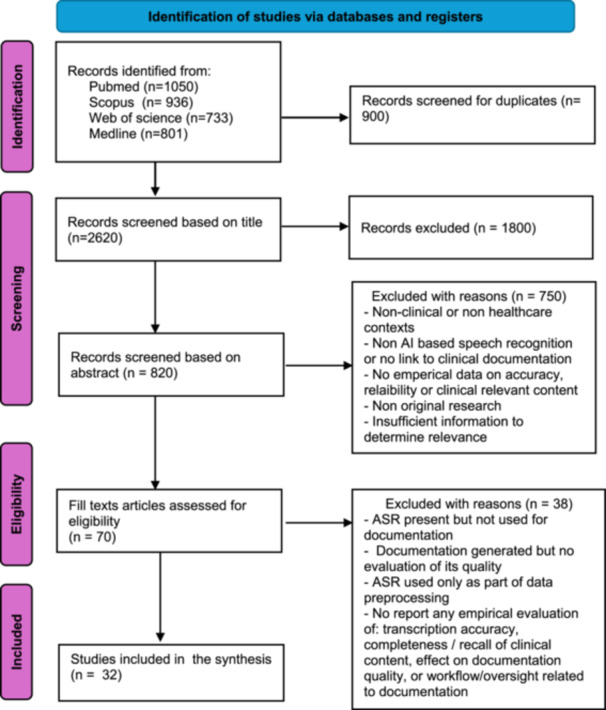
Study Selection.

The database search returned 3520 results. After the elimination of duplicates, 2620 titles were screened. A total of 820 abstracts were reviewed, and only 70 articles were selected for full‐text review. Upon full‐text review, 32 met the study eligibility criteria. Figure 1 below outlines the screening process and reasons for exclusion. Table 1 below identified key findings across the synthesised themes.

**Table 1:** Key findings across the synthesised themes.

Key theme	Types of metrics reported	Nature of findings (Key points)
System Performance and Accuracy Limitations	Word error rate (WER); report‐level error frequency; comparison vs transcription; accuracy by system; speed vs typing comparisons	Reported accuracy varied markedly across settings, from relatively low error rates in structured dictation to substantially higher rates in emergency, psychiatric, and nursing contexts. Commercial systems such as Dragon Medical, Google Cloud Speech‐to‐Text, Amazon Transcribe Medical, and Naver Clova frequently demonstrated higher accuracy than open‐source models (e.g., Whisper, Wav2Vec2), yet none achieved error‐free documentation without human correction.
Linguistic and Technical Recognition Challenges	NLCS recognition accuracy; deletion/substitution/insertion analysis; terminology and abbreviation accuracy; disparity analysis; structured vs categorical field accuracy	High misrecognition and deletion rates for short conversational cues (e.g., affirmative/negative utterances) and frequent errors in medication names, abbreviations, numbers, and proper nouns. Differential performance associated with speaker characteristics, including racial disparities and increased errors in non‐native English dictation.
Workflow Efficiency, Editing Burden, and Human Oversight	Documentation time; time‐to‐close note; editing duration; revision distance; user satisfaction; perceived vs audited accuracy; workload measures	Some AI documentation systems, including ambient scribes (e.g., DAX Copilot, Abridge) and structured voice tools (e.g., TORTUS AI), were associated with reduced note completion time and improved perceived workflow efficiency in well‐integrated settings, although clinician oversight remained essential. Editing burden remained substantial, shifting effort from drafting to verification, with ongoing human oversight required to correct omissions, inaccuracies, tone, and factual errors.
Capture of Medically Relevant Content (Precision vs Recall)	Recall of safety‐critical language; structured field completeness; narrative detail counts; precision/recall in hybrid systems; residual clinically significant error analysis	ASR frequently omitted subtle but clinically meaningful utterances, including brief patient responses and safety‐critical psychiatric language; structured fields were sometimes less complete than manual entry, whereas narrative content could be more detailed. Hybrid ASR–NLP systems achieved high precision in structured extraction tasks but showed recall weaknesses for subtle or implied findings, with trade‐offs between completeness (recall) and correctness (precision) carrying potential safety implications.

### 2.1 Overview of Included Studies

Diverse methodological approaches were used across studies to assess the reliability and accuracy of ASR for clinical documentation, including retrospective studies (*n* = 5), observational studies (*n* = 5), experimental and interventional studies (*n* = 11), secondary data analyses (*n* = 2), evaluations of AI scribe and ambient AI systems (*n* = 5), qualitative and survey studies (*n* = 3), and one tool development study (*n* = 1). Reported error rates varied widely and were measured using different metrics across studies. Therefore, the error rate measures presented reflect those reported in the primary studies and are included to illustrate variability in performance rather than enable direct comparison between tools. Appendix A summarizes the 32 included studies and the ASR technologies evaluated, while Appendix B maps the evaluation domains of accuracy, efficiency, reliability, and safety across studies.

### 2.2 Clinical Setting Distribution

The included studies were conducted across community, primary care, hospital, and specialised clinical environments. Community and Primary Care Settings (*n* = 12): One study was conducted in home, and community care [17], and eleven studies were situated in primary care or ambulatory outpatient clinics [6, 18, 19, 20, 21, 22, 23, 24, 25, 26]. Hospital‐Based Settings (*n* = 16): Four studies were conducted in general inpatient hospital settings [27, 28, 29, 30], five studies were conducted in radiology departments [4, 31, 32, 33, 34], four studies took place in emergency departments [27, 35, 36, 37]. One study was conducted in mental and behavioural health settings [38], and one in urology settings [39]. Other Settings (*n* = 3): Two studies were conducted in simulated or controlled academic environments [28, 40], and one study involved multi‐site outpatient and health system–wide implementation [41].

### 2.3 Geographic Distribution

Studies were conducted in the United States (*n* = 18), Canada (*n* = 2), and one joint Canada–United States study (*n* = 1), as well as South Korea (*n* = 2), Australia (*n* = 3), Germany (*n* = 2), the United Kingdom (*n* = 1), the Netherlands (*n* = 1), Switzerland (*n* = 1), South African (*n* = 1), Iran (n = 1).

### 2.4 ASR‐Based Clinical Documentation Technologies

Four studies evaluated cloud‐based automated speech recognition (ASR) systems, including AWS/Amazon Transcribe and Google Speech‐to‐Text [14, 15, 16, 24]. One study evaluated the Naver Clova Speech Recognition API for Korean‐language clinical conversations [16]. Two studies evaluated conventional speech‐recognition systems used for clinical documentation, including Dragon Medical One and an in‐department radiology speech‐recognition system [17, 29]. Three studies evaluated ASR‐enabled ambient AI systems such as Nuance Dragon Ambient eXperience (DAX Copilot) [20, 21, 22], and two studies evaluated the Abridge ambient AI platform [19, 39]. Individual studies also evaluated TORTUS AI [37] and Autoscriber [38]. Two studies compared multiple commercial ASR‐enabled ambient AI scribe platforms in simulated clinical encounters [18, 27]. One study conducted a large comparative evaluation of eight commercial ASR systems, including Microsoft, Google, IBM, Azure, Nuance, Amazon, and Mozilla [24].

### 2.5 System Performance and Accuracy Limitations

ASR generated transcripts frequently contain inaccuracies. Reported word error rates ranged from 7.4% in general dictated transcripts [4] to approximately 25% in mental health settings, 71% in emergency department notes [36], and as high as 97%–98% in nursing documentation [42]. Studies conducted in radiology settings reported high error frequencies, ranging from 22% to nearly 50% of reports [18, 19] and substantially worse performance than transcription [43]. Controlled experiments further show that SR produces more errors and slows documentation by 16%–18% compared with typing [27, 37]. Commercial ASR tools tend to outperform open‐source systems such as Naver Clova SR reached 75% accuracy versus 51%–58% for Google and Amazon [20], and AWS General and Medical achieved lower WERs than Whisper and Wave2Vec [17], yet even the best‐performing systems still require significant human correction. Clinicians frequently describe ASR as inconsistent or error‐prone [22, 24], and frequent substitutions, deletions, and insertions that compromise reliability were reported [34], with microphone type offering no measurable improvement [21]. Even systems with low recognizer error rates still demand edits [30].

Despite these accuracy limitations, several studies evaluating AI scribe platforms and structured voice‐assisted documentation systems reported workflow benefits. In outpatient and inpatient contexts, AI‐generated notes and letters more frequently met predefined quality thresholds compared with traditional documentation methods [40], and clinicians reported reduced typing burden and improved capture of subjective narrative content when using AI‐assisted drafting tools [26]. In these studies, editors typically refined AI‐generated drafts with relatively modest additions or deletions (approximately 45 words on average) to improve clarity and completeness [28]. Evaluations of specific AI scribe platforms described outputs as “good” to “excellent,” although not error‐free [23]. Similarly, structured voice interfaces in specialised settings were reported to outperform paper‐based or conventional electronic workflows in terms of documentation speed [44]. However, across systems, efficiency gains remained contingent on continued human oversight due to persistent recognition and factual errors.

### 2.6 Linguistic and Technical Recognition Challenges

Several studies reported limitations of ASR systems related to linguistic complexity, speaker characteristics, and technical constraints, particularly when handling short, ambiguous, or clinically significant language [17, 34, 36, 38]. For example, non‐lexical conversational sounds (NLCS), such as “mm‐hm” (yes) and “uh‐uh” (no), are crucial in clinical dialogue; however, evaluations of Google and Amazon systems reported misrecognition in over 90% of cases, typically through deletion or distortion [19]. Similar challenges appear with fillers, repetitions, short common words, and proper nouns: commercial systems frequently misheard or even “invented” filler words, with disproportionately higher error rates for Black speakers, highlighting racial performance disparities [17]. Medical terminology and abbreviations also proved difficult, as medication names such as metoprolol succinate were almost never transcribed correctly, and everyday proper nouns including “Sandy” and “Pennsylvania” were frequently misrecognized [17, 34]. Also, short, high‐frequency words, numbers, and abbreviations were reported as common sources of transcription errors [6].

ASR systems showed characteristic patterns of deletion, substitution, and insertion errors, all of which undermined clinical accuracy. In radiology, the most common ASR mistakes involve laterality confusions (left/right) and descriptor substitutions such as “one” for “none” [31], while emergency documentation demonstrates high rates of pronunciation‐related errors followed by deletions and added words [36]. Deletions and insertions also dominated ASR error patterns in dictated clinical notes [4]. Speaker‐related variability further affects performance, though the direction and magnitude vary by setting. Radiologists speaking English as a second language were more likely to produce erroneous ASR generated transcripts [43], whereas no gender or speaker‐type differences appeared in psychotherapy transcripts, where error rates were nearly identical across patients, therapists, men, and women [38]. Even within the same clinical service, surgeons demonstrated lower ASR error rates than other physicians, suggesting that speaking style or vocabulary familiarity may influence accuracy [4, 35]. Technical and system integration factors also hinder ASR reliability. Category‐based entries, such as yes/no responses or coded triage fields, were recognized far less accurately than continuous numeric data [35], and ASR systems generated numerous interface and EHR integration errors, such as misplaced text or formatting problems that were absent in typed input [27].

### 2.7 Workflow Efficiency, Editing Burden, and Human Oversight

AI tools for clinical documentation have shown mixed effects on workflow: they can speed up initial note creation but consistently impose a substantial editing burden. Clinicians frequently correct errors in ASR generated transcripts and and may avoid full dictation due to mistrust. Independent evaluations have reported higher error rates than users perceive, revealing a gap between perceived and observed ASR reliability [21]. Similar patterns of misrecognition and structural errors prompted frustration with certain ASR systems, such as VGEENS, where users especially residents found that frequent corrections and the need to dictate formatting commands diminished potential time savings and sometimes made typing preferable [22]. Despite these frustrations, efficiency gains were evident in settings where ASR tools were well‐integrated and aligned with clinical workflow. In outpatient consultations, AI‐based documentation reduced note completion time by approximately 26% without sacrificing clinician–patient interaction, and users described the system as “quick” and “easy to use,” reporting higher satisfaction among both providers and families [40]. Similar studies reported that ASR can improve documentation speed in settings such as emergency triage and ICU workflows, but often shifts effort from drafting to editing and verification, requiring clinicians to spend additional time reviewing to maintain accuracy [6, 30, 35, 44, 45].

Human oversight remains essential when using AI tools to ensure clinical accuracy [22, 40]. Although these systems produce generally usable, well‐structured drafts, none are error‐free, and clinicians routinely revise omissions, grammatical issues, and inaccuracies, particularly in Assessment and Plan (A&P) sections [23]. Over time, editing demands declined as clinicians adapted and systems improved, but accuracy still depended heavily on human review to ensure completeness, safety, and professional tone [46]. Studies consistently reinforce this need for sustained intervention, noting that human oversight is crucial not only for correcting errors in ASR‐generated transcripts but also for mitigating cognitive biases, such as confirmation bias, that may allow mistakes to persist in final documentation [22, 32, 40].

### 2.8 Capture of Medically Relevant Content (Precision vs. Recall)

Recall limitations were reported with the use of ASR, indicating that it frequently fails to capture all the details clinicians verbally provide during documentation [38, 47]. In psychiatric settings, ASR missed about one in five depression‐related terms, and error rates climbed even higher for safety‐critical utterances such as those involving self‐harm [38]. In emergency triage settings, most standard fields were more completely documented through manual entry than with the ASR tool, although the ASR tool demonstrated higher completion rates for narrative elements such as additional chief concerns and past medical history [35]. Conversely, a randomized trial reported that voice‐generated inpatient notes contained more clinical details on average, including 1.34 more history of present illness components and 1.62 more review of systems components compared to typed notes [47]. Similar studies reported that ASR accurately identified core clinical terms and avoided major errors involving names, numbers, or medications, yet frequently omitted short or subtle details such as brief patient responses (“don't know,” “can't do”) or shorthand medical expressions like “BP” for blood pressure, thereby reducing overall completeness [19, 34]. These omissions created transcripts that appeared detailed but contained critical gaps, particularly with drug names and key clinical descriptors, posing risks for documentation accuracy and patient safety [34].

Studies also reported that ASR is prone to precision errors such as incorrect insertions, substitutions, or misrecognitions that can change the meaning of documentation [4, 31, 36, 43]. Reported across studies were small misrecognitions, such as incorrect laterality or negation, carry potentially substantial consequences for patient care, and these issues persist even in structured ASR environments designed to reduce such errors [31, 36, 43]. Findings from Zhou et al. (2018) further underscore this concern, showing that insertions and substitutions continue to appear in ASR documents and that a notable share of remaining errors after review are clinically significant [4]. Such results raise concerns about the intrinsic limits of current ASR systems in consistently producing safe, high‐fidelity clinical text without oversight. Conversely, more advanced ASR–NLP hybrid systems demonstrated strong performance, achieving precision levels around 96% in structured extraction tasks, but they still displayed recall weaknesses, particularly for subtle or implied findings [32, 33].

## 3 Discussions

This review synthesised evidence across diverse clinical settings examining the performance, limitations, and workflow impact of ASR systems. The included studies provided substantial quantitative and qualitative evidence relating to accuracy constraints, linguistic vulnerability, workflow consequences, and the need for ongoing human oversight. Synthesising the evidence, current ASR tools appear most appropriate when implemented as assistive drafting systems rather than autonomous solutions. Across studies, optimal use depends on:

- Structured human review processes to identify deletions, substitutions, and clinically significant misrecognitions.
- Workflow integration that enables verification without introducing excessive cognitive burden.
- Awareness of linguistic and demographic performance variability, particularly for short affirmative cues, medication names, numbers, and safety‐sensitive language.
- Monitoring of recall limitations to prevent omission of subtle but clinically meaningful information.

Although ASR generated transcripts can enhance drafting speed and structural organisation, recognition errors remain frequent, particularly in emergency, mental health, radiology, and nursing contexts. Editing and verification demands were consistently reported, and independent audits often identified more errors than users perceived, revealing a trust–performance gap. Overall, system value appears contingent on socio‐technical alignment, sustained oversight, and evaluation beyond aggregate accuracy metrics to include safety, completeness, and equity considerations.

### 3.1 What This Study Adds to the Literature

Our main finding is that AI‐based speech recognition systems remain assistive rather than autonomous documentation tools. While clinicians report reduced typing burden, improved workflow flow, and enhanced patient engagement, the evidence consistently demonstrates that recognition accuracy remains insufficient for unsupervised clinical use. Across clinical settings, studies reported variable performance of ASR systems, with substantial differences in error rates across contexts [4, 27, 48]. For example, error rates substantially exceed those of professional transcription, and clinically significant misrecognitions, particularly involving medication names, measurements, negation, and laterality, persist across settings. A previous evaluation of ASR‐generated notes found a word error rate of approximately 7.4%, compared with only 0.4% for professional transcription; many of these errors were clinically significant (e.g., incorrect drug names or measurements) [4]. Similar studies conducted in emergency departments reported that SR‐based documentation was more error‐prone and time‐consuming than simple typing, indicating a persistent reliability and accuracy gap in commercial ASR models [27, 48].

Linguistic complexity further contributes to error vulnerability. Contemporary ASR systems struggle with brief utterances, conversational cues, and complex medical terminology. More than 94% of “yes/no” cues were misrecognized or omitted in some evaluations, and medication names were frequently mistranscribed or deleted [5, 49]. Although such errors may appear minor, they can alter meaning or remove clinically relevant nuance. While AI documentation tools may reduce typing effort, they can simultaneously increase cognitive workload due to the need for verification and editing. Observational audits reveal that ASR errors appear in a far higher proportion of notes than users perceive, indicating a gap between clinician trust and actual system performance [50, 51]. These findings suggest that current ASR systems remain unsuitable for unsupervised documentation and require substantial post‐transcription review to meet clinical quality standards.

Beyond aggregate performance metrics, several included studies explicitly examined demographic and linguistic performance disparities in ASR systems. Large‐scale comparative evaluations demonstrated significantly higher word error rates for Black speakers compared with white speakers, indicating measurable racial performance gaps in commercial ASR platforms [52]. Another study, using a retrospective chart review of radiology reports, identified elevated transcription error rates among clinicians dictating in English as a second language, suggesting that accent and phonetic variation remain persistent sources of differential system performance [29]. Importantly, these disparities were not confined to global accuracy measures. Qualitative and quantitative studies examining word error rates in ASR systems and their potential impact on the quality of clinical documentation have reported systematic deletion or misrecognition of short conversational cues, non‐lexical utterances, and brief affirmative or negative responses [19, 50]. Such linguistic elements, although brief in duration, frequently carry critical clinical meaning. In psychiatric contexts, study identified disproportionate recall errors for depression‐related terminology and self‐harm expressions, indicating potential amplification of risk in safety‐sensitive domains [38].

The implications extend beyond transcription fidelity. A qualitative study on impact of ASR errors on African Americans linked higher correction burdens to increased cognitive workload, raising concerns that clinicians from underrepresented linguistic or racial backgrounds may experience disproportionate editing demands [52]. In multi‐provider settings, studies highlighted how inconsistent documentation quality may undermine continuity of care and record reliability [17]. In high‐risk environments such as emergency medicine and mental health services, systematic misrecognition of subtle language may amplify patient safety vulnerabilities [27, 38, 50], a concern echoed by patient safety bodies that caution speech recognition–related documentation errors can introduce downstream harm when verification processes are insufficient [53].

Collectively, these findings posit that AI‐based documentation systems should be positioned as assistive rather than autonomous tools [4]. Developers should prioritise improving recall performance and reducing deletion and negation errors, particularly for safety‐critical language and medication names, which remain vulnerable to misrecognition [21]. Training datasets must be demographically and linguistically representative, with routine bias auditing and stratified performance reporting, as racial disparities in ASR accuracy have been empirically demonstrated [17, 54]. Clinicians and organisations should embed structured verification protocols into workflow, particularly in high‐risk settings, to mitigate automation bias and overreliance on AI outputs [54]. Independent evaluations of AI scribes further highlight the need for sustained human oversight and monitoring of editing burden [13, 55, 56]. Policymakers should extend evaluation standards beyond aggregate word error rates to include recall, clinically significant errors, demographic disparities, and real‐world workload impact [21, 57].

### 3.2 Implications for Future Research

The heterogeneity observed across evaluation approaches underscores the need for greater standardisation in future ASR research. To improve comparability and clinical relevance, evaluations should adopt a multidimensional framework that extends beyond aggregate word error rate alone. Future studies should report context‐specific transcription accuracy stratified by clinical setting and encounter type, alongside explicit measurement of clinically significant errors, particularly those involving negation, laterality, medication names, and numerical values. Precision and recall should be assessed separately to capture both incorrect insertions and omissions of safety‐critical or subtle language.

Workflow impact should be measured using objective indicators such as time to complete documentation, editing duration, and post‐edit residual error rates, rather than relying solely on subjective perceptions of efficiency. Given documented variability across speakers and contexts, demographic and linguistic stratification should be routinely incorporated to assess equity in system performance. Finally, evaluation frameworks should include structured measures of human oversight burden, including verification workload and discrepancies between perceived and observed accuracy. Systematic reporting across these domains would support more meaningful cross‐study comparison and provide a clearer basis for assessing the clinical readiness, safety, and equity of ASR systems in healthcare documentation.

### 3.3 Study Strength and Limitations

This scoping review provides a focused synthesis of reported accuracy, reliability, efficiency, and clinical information capture of ASR systems across diverse healthcare settings. By integrating quantitative performance metrics and qualitative user‐reported experiences, the review offers a multidimensional overview of how ASR performance has been evaluated. However, substantial heterogeneity in study designs, clinical contexts, system types, and performance metrics (e.g., word error rate, precision/recall, documentation time, user‐reported burden) limited the ability to conduct direct cross‐study comparisons or pooled quantitative synthesis. The review, therefore, maps reported evidence rather than generating comparative effect sizes or performance rankings. Additionally, the evidence base is skewed toward English‐language studies from high‐income health systems, limiting generalisability to multilingual or resource‐limited settings.

## 4 Conclusion

Our scoping review shows that AI‐based speech recognition can improve documentation and capture many spoken details, sometimes even more history, yet it consistently misses or distorts important utterances and exhibits systematic bias. Error rates reported in the literature indicate that up to seventy percent of notes generated by ASR contain errors, far exceeding acceptable quality thresholds. These limitations suggest that, although AI speech recognition tools reduce documentation burden and improve workflow efficiency when well integrated, they must remain tools used with consistent human oversight rather than replacements for clinician‐authored documentation. As emphasized across study findings, ongoing evaluation and refinement of ASR and NLP technologies are essential, but expectations should be tempered by the inherent constraints these systems face in achieving the precision and completeness required for safe clinical practice.

## Author Contributions

The scoping review was conceptualized by O.O., while screening of articles for inclusion and exclusion and data extraction was conducted by O.O. and A.S. The drafted manuscript was critically reviewed by O.O., A.S., and K.O. prior to submission. All authors read and approved the final manuscript.

## Funding

The authors have nothing to report.

## Ethics Statement

The authors have nothing to report.

## Conflicts of Interest

The authors declare no conflicts of interest.

## Data Availability

Data sharing not applicable to this article as no datasets were generated or analysed during the current study.

## Appendix A Study characteristics

**Table:**

Study	Country	Aim/Objective	Design	Setting & specialty	Participants/Sample	Technology/System(s)	Data source	MMAT scores in %
Zolnoori et al., 2024 [17]	United States (NYC)	Evaluate accuracy of four ASR systems in transcribing patient–nurse communication and assess racial disparities between Black and White patients.	Observational comparative evaluation using real‐world recordings.	Home healthcare (VNS Health); community nursing.	35 patients (16 Black, 19 White); 860 utterances.	AWS Transcribe (General/Medical), OpenAI Whisper (Large V3), Meta Wav2Vec 2.0.	Real audio recordings of home visits.	100%
Tran et al., 2023 [19]	United States (CA & MI)	Assess impact of non‐lexical conversational sounds on ASR performance.	Empirical evaluation of two ASR engines.	Ambulatory primary care.	36 reenacted encounters; 135k spoken words.	Google Speech‐to‐Text Clinical, Amazon Transcribe Medical.	Studio dataset of primary‐care dialogues.	75%
Lee et al., 2022 [20]	South Korea (Seoul)	Evaluate ASR accuracy for Korean medical terms in doctor–patient conversations.	Comparative evaluation of three ASR APIs.	Cardiology outpatient clinic.	112 adult patients.	Naver Clova SR, Google Speech‐to‐Text, Amazon Transcribe.	Real consultation audio.	75%
Blackley et al., 2020 [21]	United States (Boston MA)	Compare EHR note quality and efficiency using speech recognition vs typing.	Controlled within‐subject study.	Primary care (simulated Epic notes).	10 physicians experienced with Dragon.	Dragon Medical One (Nuance).	Simulated encounters documented in Epic.	100%
Kodish‐Wachs et al., 2018 [34]	United States (PA)	Compare eight ASR engines for clinical speech accuracy and concept capture.	Experimental comparison (simulated interviews).	Primary care (simulated CMS cases).	11 scenarios; 2 physicians + 7 lay participants.	Eight ASRs (Microsoft, Google, IBM, Azure, Nuance, Amazon, Mozilla).	Simulated audio recordings.	100%
Quint et al., 2008	United States (MI)	Determine frequency of speech‐recognition errors in final radiology reports.	Retrospective review of finalized reports.	Radiology – Thoracic oncology.	265 reports by 41 radiologists.	In‐department SR system.	Finalized radiology reports.	75%
Albrecht et al., 2025 [22]	United States (Kansas City KS)	Evaluate impact of ambient AI (Abridge) on clinician experience and burden.	Pre/post implementation survey.	Multispecialty ambulatory clinics.	181 clinicians (30 specialties).	Abridge AI platform integrated with Epic.	Clinician surveys (analyzed in R).	100%
Balloch et al., 2024 [40]	United Kingdom (London)	Assess ambient AI tool impact on consultation experience and documentation quality.	Controlled simulation study.	Outpatient specialty clinics (GOSH).	8 clinicians, 6 actors, 47 consultations.	TORTUS AI (ASR + GPT‐4 summarization).	Simulated consultations with mock EHR.	100%
Van Buchem et al., 2024 [28]	Netherlands (Leiden & Utrecht)	Assess impact of Dutch digital scribe on documentation time and quality.	Usability and comparative evaluation of manual vs automatic vs edited summaries.	Simulated internal medicine consultations.	21 medical students, 27 recordings (430 summaries).	Autoscriber (ASR + GPT‐3.5/4 summarization).	Mock consultations (Leiden UMC).	100%
Ha et al., 2025 [23]	Canada & United States (Toronto)	Systematically compare usability, technical performance, and accuracy of AI scribes for primary care.	Competitive cross‐system comparative analysis.	Primary care (simulated encounters, standardized cases).	Six AI scribes tested using four standardized 15‐min audio encounters.	Six commercial AI scribe platforms (ASR + NLP; anonymized).	Simulated 15‐min primary care audios; verbatim transcripts & SOAP notes as reference.	100%
Afshar et al., 2025 [41]	United States (Madison, WI)	Develop and evaluate a framework for safe implementation, monitoring, and evaluation of ambient AI documentation systems (PTOps Playbook).	Implementation framework + stepped‐wedge pragmatic randomized controlled trial.	Multispecialty outpatient and ambulatory clinics (UW Health).	66 providers (20 in initial rollout; 8,527 encounters; ongoing 24‐week RCT).	Abridge Ambient AI integrated with Epic (FHIR APIs + Haiku app); LLM coder for ICD‐10.	Real‐world EHR & REDCap data; QlikView dashboards; open‐source GitLab materials.	75%
Shah et al., 2025 [24]	United States (Stanford, CA)	Explore physician perspectives on AI scribes, focusing on adoption, workflow, and experience, guided by RE‐AIM/PRISM framework.	Qualitative study (semistructured interviews + thematic analysis).	Ambulatory primary care and specialty practices.	22 physicians (13 primary care, 9 specialty); 12 community, 10 faculty practices; 3‐month pilot (Nov 2023–Jan 2024).	Nuance Dragon Ambient eXperience Copilot (Microsoft), integrated with Epic.	Transcribed Zoom interviews analyzed in Dedoose 9.2.4 (RE‐AIM/PRISM).	75%
Bundy et al., 2024 [25]	United States (North Carolina)	Evaluate physicians' experiences with DAX Copilot and its effects on administrative and cognitive burden.	Qualitative study (semi‐structured interviews + thematic analysis).	Academic primary care clinics (internal medicine, family medicine, pediatrics).	12 physicians purposively sampled (from 116 DAXC pilot users).	Nuance Dragon Ambient eXperience Copilot (GPT‐4 based).	Semi‐structured Teams interviews; coded and analyzed in ATLAS. ti and Miro.	100%
Moryousef et al., 2025 [39]	Canada (McMaster & Western University)	Evaluate AI scribe performance and perceived quality in urology clinical contexts.	Cross‐sectional experimental evaluation with blinded survey.	Outpatient urology consultations (academic centers).	20 urologists (faculty + trainees).	Nabla, Tali, Heidi, ScribeMD, and Scribeberry AI scribes.	Simulated audio consultations (urolithiasis, BPH, PSA); Google Forms survey.	100%
Duggan et al., 2025 [26]	United States (Philadelphia, PA)	Evaluate impact of ambient scribing on documentation efficiency, burden, and clinician experience.	Prospective single‐group pre‐post quality improvement study.	Multispecialty outpatient clinics (17 specialties).	46 clinicians (40 MDs, 4 PAs, 2 NPs); mean 11.1 years practice.	Nuance Dragon Ambient eXperience (DAX Copilot) integrated with Epic (Haiku).	EHR Signal analytics + pre/post surveys with qualitative feedback.	75%

Author & Year	Country	Aim/Objective	Design	Setting & Specialty	Participants/Sample	Technology/System(s)	Data Source	MMAT Scores in %
Goss et al., 2016 [50]	United States	To determine the incidence and types of SR errors introduced by this technology in the emergency department (ED).	A random sample of 100 notes was annotated. Two board certified EPs completed error analysis.	Level 1 emergency department in a tertiary academic teaching hospital.	100 notes dictated by attending emergency physicians between January and June 2012.	“Dragon Medical Software 10.0 or 10.1” and “Nuance PowerMic II.”	ED electronic health record dictated notes.	100%
du Toit et al., 2015 [43]	South Africa	To compare the accuracy of SR and DT reports in a resource limited setting.	The first 300 SR and the first 300 DT reports were reviewed by a single observer.	Tygerberg Hospital, Department of Diagnostic Radiology.	300 SR and 300 DT reports with a follow up analysis 4 years later.	Speech recognition (SR) and traditional dictation transcription (DT).	Radiology PACS reports.	75%
Sippel et al., 2023 [18]	United States	To characterize patient perceptions of SRIER.	Exploratory survey administered to 65 consecutive patients.	Internal medicine and pulmonary medicine clinics and a community family practice clinic.	65 consecutive patients receiving acute, chronic, and wellness care.	Dragon Medical One and Dragon Powermic III.	After visit summaries and patient surveys.	75%
Lybarger et al., 2018 [30]	United States	To understand how physicians use ASR to create clinical notes and the characteristics of the edits.	Analysis of a corpus of 649 dictated clinical notes comparing ASR transcripts and final edited notes.	Inpatient progress notes in internal medicine services at two teaching hospitals.	649 dictated clinical notes from 9 physicians.	Dragon Medical Edition 2, VGEENS, and portable wireless recording devices.	ASR transcripts and final edited notes.	75%
Schmidt et al., 2024 [32]	Australia	To evaluate the performance of five generative LLMs in detecting speech recognition errors in radiology reports.	Retrospective study using 3233 de identified radiology reports with manual error detection as the reference.	Major tertiary hospital providing CT and MRI radiology reporting.	3233 radiology reports produced by 22 attending physicians and 23 residents.	“GPT‐4,” “GPT‐3.5‐turbo,” “text‐davinci‐003,” “Llama v2‐70B‐chat,” “Bard.”	Picture archiving and communication system containing de identified radiology reports.	75%
Shour et al., 2025 [6]	United States	To analyze the association between SR tool usage and documentation efficiency.	Cross sectional study following STROBE guidelines.	Marshfield Clinic Health System in rural settings across various specialties.	1124 clinicians with a mean age of 47.68 years, 52 percent female.	“Dragon Medical One.”	Retrospective documentation efficiency metrics.	100%
Payne et al., 2018 [45]	United States	To determine if note timeliness, quality, and physician satisfaction are improved.	Randomized controlled trial.	Inpatient general medicine at the University of Washington Medical Center and Harborview Medical Center.	709 intervention notes and 1143 control notes.	“VGEENS” and “Dragon Medical Practice Edition.”	EHR notes from the randomized controlled trial.	75%
Hodgson et al., 2017 [27]	Australia	To compare the efficiency and safety of SR assisted clinical documentation with keyboard and mouse.	Within subject experimental study using randomly allocated tasks.	Emergency department clinicians using a commercial EHR.	35 emergency department clinicians.	“Nuance Dragon Medical 360” and “Cerner Millennium FirstNet.”	Simulated documentation tasks and EHR logs.	75%
Vosshenrich et al., 2021 [31]	Switzerland	To investigate the most common errors in residents' preliminary reports, the impact of structured reporting, and implications for patient safety.	Retrospective study at a single institution.	Radiology including neuroradiology, musculoskeletal, CT, body, breast, and nuclear medicine.	78,625 radiology reports produced between September 2017 and December 2018.	“SpeechMagic 8” and “Centricity RIS‐i 6.0.”	Institutional RIS containing preliminary and final radiology reports.	75%
Peivandi et al., 2022 [42]	Iran	To evaluate the use of online and offline speech recognition software on spelling errors in nursing reports.	Interventional study using a crossover design.	Inpatient wards of three educational hospitals covering 10 specialties.	70 nurses assigned to two groups of 35.	“Nevisa 3.2,” “Speechtexter,” and “Andrea NC‐8 microphone.”	Nursing admission reports.	100%
Miner et al., 2020 [38]	United States	To quantify the accuracy of automatic speech recognition including overall accuracy, detection of depression specific language, and harm related conversations.	Secondary analysis of 100 therapy sessions from a randomized controlled trial.	23 college counseling sites providing psychotherapy.	100 patients and 78 therapists. Average session length 45 min.	“Google Cloud Speech to Text.”	Audio recordings and human transcripts.	75%
White et al., 2020 [47]	United States	To measure the difference in professional fee charges between using voice and keyboard.	Randomized trial with secondary analysis.	Inpatient general medicine services.	1,613 notes created by 31 physicians.	“VGEENS” and “Dragon Medical Practice Edition.”	EHR notes with blinded independent coding.	100%
Cho et al., 2022 [35]	South Korea	To investigate the promptness and reliability of a real time record input assistance system and compare it to manual triage.	Prospective interventional study.	Level 1 emergency department triage unit in northwestern Seoul.	1063 triage tasks performed by 19 nurses.	“RMIS‐AI” real time STT and NLP system developed by Selvas AI.	Triage records generated manually and through RMIS AI.	100%
Zhou et al., 2018 [4]	United States	Analysis of errors in dictated clinical documents assisted by speech recognition software and professional transcriptionists.	Retrospective analysis		Not stated	Speech recognition software and professional transcriptionists.	Dictated clinical documents	75%
Peine et al., 2023 [44]	Germany	To compare established documentation and retrieval processes with a Voice Information and Documentation System (VIDS) regarding performance, accuracy, mental workload, and user experience.	Crossover study with randomized order of system exposure.	Intensive Care Units (ICUs); documentation and patient data management.	60 ICU‐experienced medical professionals (physicians, ICU nurses, advanced medical students).	Paper‐based documentation (“MEDLINQ Curve”), PDMS (“IntelliSpace Critical Care & Anesthesia”), VIDS (“Mona” by Clinomic GmbH)	Fictitious ICU patient records; performance metrics; NASA‐TLX and meCUE2.0 questionnaires; expert‐validated gold standard reco	100%
Jorg et al., 2023 [33]	Germany	To use the potential of NLP to combine the advantages of SR and speech recognition.	Development and evaluation of a novel reporting tool; testing with fictitious samples and original reports.	Radiology; urolithiasis CT reporting.	82 fictitious text samples and 50 original free‐text CT reports authored by 13 board‐certified radiologists.	Indicda® speech; Empolis Knowledge Express®; Microsoft®. NET application; RadLex ontology; task‐oriented dialogue system.	Fictitious text examples created by a radiologist and 50 original CT free‐text reports manually extracted from imaging archive.	100%
Hodgson et al., 2018 [37]	Australia	To validate previously identified significant risks and inefficiencies associated with the use of speech recognition (SR) for documentation within an electronic health record (EHR) system.	A within‐subject experimental study replicating the methods of a previous experiment (Experiment 1).	Emergency department.	Thirty‐five emergency department clinicians from four urban teaching hospitals in Sydney, Australia.	Cerner Millennium suite with the FirstNet ED component (v2015.01.11) and Nuance Dragon Medical 360 Network Edition (UK) (v2.4.2	Recorded participant sessions, EHR system logs standardized clinical documentation tasks.	75%

## Appendix B Mapping of Accuracy, Efficiency, Reliability, and Safety Evaluation Domains Across Included Studies

**Table:**

Study (Year)	Setting/Context	System(s)	Transcription accuracy metrics	Efficiency outcomes	Reliability/safety indicators	Safety‐relevant error types highlighted	Demographic stratification
Zolnoori et al. (2024) [17]	Home healthcare nurse–patient communication	AWS Transcribe (General/Medical), Whisper v3, Wav2Vec2	Comparative accuracy/WER (by system)	Not reported (NR)	Disparity‐focused reliability assessment	Deletions/substitutions; clinically meaningful utterance loss	Yes (Black vs White patients)
Tran et al. (2023) [19]	Ambulatory primary care dialogues (NLCS focus)	Google STT Clinical, Amazon Transcribe Medical	Accuracy of NLCS; high misrecognition/deletion rates	Perceived time/workload burden	Reliability of conversational cue capture	Deletion/misrecognition of “mm‐hm/uh‐uh” yes/no cues	No
Lee et al. (2022) [20]	Korean cardiology outpatient conversations	Naver Clova, Google STT, Amazon Transcribe	Comparative accuracy for medical terms	NR	Reliability via term‐level performance	Misrecognition of medical terminology	No
Kodish‐Wachs et al. (2018) [34]	Simulated conversational clinical speech	8 ASR engines (incl. Microsoft/Google/IBM/Nuance/Amazon etc.)	Comparative accuracy + concept capture	NR	Reliability via error typology + concept capture	Substitutions/deletions; missed concepts	No
Zhou et al. (2018) [4]	Dictated clinical documents vs transcription	SR software vs professional transcription	Error rate/WER‐like measures; error classification	NR	Clinically significant residual errors after review	Medication/measurement errors; deletions/insertions/substitutions	No
Goss et al. (2016) [50]	Emergency department dictated notes	Dragon Medical + PowerMic	Incidence of errors in ED notes	NR	Critical/clinically significant errors	ED documentation errors; risk‐relevant misrecognitions	No
Miner et al. (2020) [38]	Psychotherapy sessions	Google Cloud STT	Overall accuracy/WER; depression language detection	NR	Safety‐relevant recall limitations	Missed depression/self‐harm expressions; recall errors	No
Peivandi et al. (2022) [42]	Nursing documentation	Nevisa, Speechtexter (online/offline)	Error frequency (spelling/transcription errors)	NR (crossover compares modalities)	Reliability in nursing notes quality	High error rates in nursing reports	No
du Toit et al. (2015) [43]	Radiology in resource‐limited setting	SR vs traditional dictation transcription	Accuracy comparison SR vs DT	NR	Reliability of final reports	Misrecognitions in radiology reports	No (multilingual context noted)
Quint et al. (2008) [29]	Radiology final reports	In‐department SR	Error frequency/spectrum	NR	Reliability of finalised reports	Radiology reporting errors (varied)	No
Vosshenrich et al. (2021) [31]	Radiology proofreading data mining	SpeechMagic + RIS	Error mining frequency patterns	NR	Patient‐safety implications via error types	Laterality, negation‐like and descriptor errors	No
Schmidt et al. (2024) [32]	Radiology error detection	GPT‐4, GPT‐3.5, etc.	Detection performance vs manual reference	NR	Reliability of automated error detection	SR error detection in radiology reports	No
Jorg et al. (2023) [33]	Radiology structured reporting	Indicda speech + NLP + ontology	Accuracy in structured extraction/precision‐recall style	NR	Reliability of structured reporting workflow	Subtle/implicit findings missed (recall limits)	No
Peine et al. (2023) [44]	ICU documentation & retrieval	“Mona” voice system vs PDMS vs paper	Accuracy vs gold standard record	Task performance/workload (NASA‐TLX)	Safety via workload + accuracy	Errors affecting ICU data entry/retrieval	No
Cho et al. (2022) [35]	ED triage voice AI	RMIS‐AI (STT + NLP)	Reliability/accuracy of structured fields	Triage promptness/task performance	Reliability of categorical entry capture	Lower accuracy for categorical yes/no vs continuous numeric	No
Hodgson et al. (2017) [27]	ED EHR documentation	Dragon Medical 360 + Cerner FirstNet	Accuracy/safety comparison SR vs keyboard	Time/efficiency outcomes	Safety assessment of SR in EHR	Interface/integration errors; clinically meaningful substitutions	No
Hodgson et al. (2018) [37]	ED replication study	Same as above	Accuracy/safety replication outcomes	Task completion time	Safety risk replication	Similar SR safety/efficiency risks	No
Blackley et al. (2020) [21]	Simulated primary care EHR notes	Dragon Medical One	Errors vs typing; note quality	Efficiency comparison SR vs typing	Reliability gap between perceived vs actual	Substitutions/deletions; formatting‐type issues	No
Payne et al. (2018) [45]	Inpatient progress notes	VGEENS + Dragon	Note quality/timeliness + satisfaction	Timeliness outcomes	Reliability via note quality measures	Errors/quality issues in inpatient notes	No
White et al. (2020) [47]	Inpatient progress notes + billing	VGEENS + Dragon	Indirect documentation quality (coding differences)	NR	Reliability via coding/clinical detail differences	Content differences affecting coding/interpretation	No
Lybarger et al. (2018) [30]	Physician editing of dictated notes	Dragon + VGEENS	Corpus comparison: raw ASR vs final note	Editing burden (edit distance/edits)	Reliability through post‐edit residual errors	Persistent errors after editing	No
Shour et al. (2025) [6]	Multi‐specialty system‐wide use	Dragon Medical One	NR (usage association study)	Documentation efficiency metrics (EHR logs)	Reliability inferred via workflow metrics	NR	No
Sippel et al. (2023) [18]	Patient perceptions (exam room)	Dragon Medical One	NR	NR	Reliability via patient acceptability	NR	No
Albrecht et al. (2025) [22]	Multispecialty ambulatory	Abridge ambient AI	NR (survey‐based)	Burden/burnout proxies; perceived efficiency	Reliability via clinician perception	NR	No
Duggan et al. (2025) [26]	Multispecialty outpatient	DAX Copilot + Epic	NR (QI study focus)	Time/burden outcomes (pre–post)	Reliability via clinician experience + oversight needs	Errors requiring verification	No
Shah et al. (2025) [24]	Physician perspectives	DAX Copilot + Epic	NR (qualitative)	Perceived efficiency/workflow impact	Reliability concerns and adoption conditions	Error‐proneness; verification behaviours	No
Bundy et al. (2024) [25]	Primary care clinics	DAX Copilot	NR (qualitative)	Cognitive/admin load effects	Reliability via trust/oversight narratives	Automation bias/overreliance risks noted	No
Afshar et al. (2025) [41]	Multi‐site pragmatic deployment	Abridge + Epic (FHIR/Haiku)	Monitoring framework (not single metric)	Operational metrics in pragmatic rollout	Safety monitoring/evaluation framework	Safety governance risks; monitoring indicators	Not primary (monitoring can support stratification)
Balloch et al. (2024) [40]	Simulated outpatient specialty clinics	TORTUS AI (ASR + GPT summarisation)	Documentation quality vs standard	~26% reduction in note completion time	Reliability via required oversight	Omissions/inaccuracies requiring review	No
van Buchem et al. (2024) [28]	Simulated internal medicine	Autoscriber (ASR + GPT)	Quality comparison manual vs auto vs edited	Documentation time outcomes	Reliability via edit requirements	Omissions/clarity edits (~word changes)	No
Ha et al. (2025) [23]	Primary care competitive analysis	6 commercial AI scribes	Cross‐system accuracy + technical performance	NR (focus on usability/performance)	Reliability via comparative outputs	Error presence; not error‐free	No (cross‐system, not demographic)
Moryousef et al. (2025) [39]	Urology outpatient scenarios	Nabla, Tali, Heidi, ScribeMD, Scribeberry	Perceived performance/quality ratings	NR	Reliability via clinician‐rated usefulness	Errors/omissions noted in ratings	No

## Appendix C Search strings across databases

**Table:**

Database	Date	Search Strategy	Records Retrieved (n)
PubMed	2015–2025	((“Speech Recognition Software”[Mesh] OR “Artificial Intelligence”[Mesh] OR “Natural Language Processing”[Mesh] OR “speech recognition”[tiab] OR “automatic speech recognition”[tiab] OR ASR[tiab] OR “voice recognition”[tiab] OR dictat*[tiab] OR “speech‐to‐text”[tiab] OR “ambient clinical documentation”[tiab] OR “ambient AI”[tiab] OR “AI scribe”[tiab] OR “digital scribe”[tiab] OR “virtual scribe”[tiab]) AND (“Medical Records Systems, Computerized”[Mesh] OR “Electronic Health Records”[Mesh] OR “clinical documentation”[tiab] OR “clinical note*”[tiab] OR “progress note*”[tiab] OR “radiology report*”[tiab] OR “discharge summar*”[tiab] OR EHR[tiab] OR “electronic health record*”[tiab]) AND (accurac*[tiab] OR error*[tiab] OR “word error rate”[tiab] OR WER[tiab] OR reliab*[tiab] OR valid*[tiab] OR completeness[tiab] OR recall[tiab] OR precision[tiab] OR “information capture”[tiab] OR “documentation quality”[tiab] OR usability[tiab] OR workflow[tiab] OR efficien*[tiab] OR “time savings”[tiab] OR “editing burden”[tiab]))	1050
Scopus	2015–2025	TITLE‐ABS‐KEY((“speech recognition” OR “automatic speech recognition” OR ASR OR “voice recognition” OR dictat* OR “speech‐to‐text” OR “ambient clinical documentation” OR “ambient AI”OR “AI scribe”OR “digital scribe”OR “virtual scribe”) AND (“clinical documentation” OR “clinical note*” OR “progress note*” OR “radiology report*” OR “discharge summar*” OR EHR OR “electronic health record*”) AND (accurac* OR error* OR “word error rate” OR WER OR reliab* OR valid* OR completeness OR recall OR precision OR “information capture” OR “documentation quality” OR usability OR workflow OR efficien* OR “time savings” OR “editing burden”)) AND PUBYEAR > 2014 AND PUBYEAR < 2026	936
Web of Science	2015–2025	TS = ((“speech recognition” OR “automatic speech recognition” OR ASR OR “voice recognition” OR dictat* OR “speech‐to‐text” OR “ambient clinical documentation” OR “ambient AI”OR “AI scribe”OR “digital scribe”OR “virtual scribe”) AND (“clinical documentation” OR “clinical note*” OR “progress note*” OR “radiology report*” OR “discharge summar*” OR EHR OR “electronic health record*”) AND (accurac* OR error* OR “word error rate” OR WER OR reliab* OR valid* OR completeness OR recall OR precision OR “information capture” OR “documentation quality” OR usability OR workflow OR efficien* OR “time savings” OR “editing burden”))	733
MEDLINE	2015–2025	1. exp Speech Recognition Software/2. exp Artificial Intelligence/OR exp Natural Language Processing/3. (speech recognit* OR automatic speech recognit* OR ASR OR voice recognit* OR dictat* OR speech‐to‐text OR ambient documentation OR ambient AI OR AI scribe* OR digital scribe* OR virtual scribe*). ti, ab. 4. 1 OR 2 OR 3 5. exp Electronic Health Records/OR exp Medical Records Systems, Computerized/6. (clinical documentation OR clinical note* OR progress note* OR radiology report* OR discharge summar* OR EHR OR electronic health record*). ti, ab. 7. 5 OR 6 8. (accurac* OR error* OR word error rate OR WER OR reliab* OR valid* OR completeness OR recall OR precision OR information capture OR documentation quality OR usability OR workflow OR efficien* OR time savings OR editing burden). ti, ab. 9. 4 AND 7 AND 8 10. limit 9 to yr = “2015–2025”	801
